# Immunobiological Outcomes of Repeated Chlamydial Infection from Two Models of Within-Host Population Dynamics

**DOI:** 10.1371/journal.pone.0006886

**Published:** 2009-09-03

**Authors:** David M. Vickers, Qian Zhang, Nathaniel D. Osgood

**Affiliations:** 1 Department of Interdisciplinary Studies, University of Saskatchewan, Saskatoon, Saskatchewan, Canada; 2 Department of Computer Science, University of Saskatchewan, Saskatoon, Saskatchewan, Canada; 3 Community Health and Epidemiology, University of Saskatchewan, Saskatoon, Saskatchewan, Canada; Singapore Immunology Network, Singapore

## Abstract

**Background:**

*Chlamydia trachomatis* is a common human pathogen that mediates disease processes capable of inflicting serious complications on reproduction. Aggressive inflammatory immune responses are thought to not only direct a person's level of immunity but also the potential for immunopathology. With human immunobiology being debated as a cause of prevailing epidemiological trends, we examined some fundamental issues regarding susceptibility to multiple chlamydial infections that could have implications for infection spread. We argue that, compared to less-frequent exposure, frequent exposure to chlamydia may well produce unique immunobiological characteristics that likely to have important clinical and epidemiological implications.

**Methods and Results:**

As a novel tool for studying chlamydia, we applied principles of modeling within-host pathogen dynamics to enable an understanding of some fundamental characteristics of an individual's immunobiology during multiple chlamydial infections. While the models were able to reproduce shorter-term infection kinetics of primary and secondary infections previously observed in animal models, it was also observed that longer periods between initial and second infection may increase an individual's chlamydial load and lengthen their duration of infectiousness. The cessation of short-term repeated exposure did not allow for the formation of long-lasting immunity. However, frequent re-exposure non-intuitively linked the formation of protective immunity, persistent infection, and the potential for immunopathology.

**Conclusions:**

Overall, these results provide interesting insights that should be verified with continued study. Nevertheless, these results appear to raise challenges for current evidence of the development of long-lasting immunity against chlamydia, and suggest the existence of a previously unidentified mechanism for the formation of persistent infection. The obvious next goal is to investigate the qualitative impact of these results on the spread of chlamydia.

## Introduction

The genus *Chlamydiae* encompasses a unique class of obligate intracellular bacteria that can cause disease in a wide range of animals [1]. Similarly to all other commonly reported sexually transmitted infections (STIs) (e.g., gonorrhea), *Chlamydia trachomatis* (*Ct*) represents a unique and important public health concern, as prevention efforts are hampered by cryptic infections and delayed diagnosis [2], [3]. This ability to thrive, despite improved understanding of the immunobiology, pathogenesis, and epidemiology of this bacterium [3], has made it the world's most common cause of sexually transmitted disease [2].

*Chlamydia* bacteria have a biphasic developmental cycle that consists of an extra- and intracellular form [1]–[2]. The extracellular form, the elementary body (EB), is infectious and thought to be metabolically static. During infection, the EB is internalized into host epithelial cells via small vacuoles resembling endosomes, most of which avoid fusion with host cell lysosomes [2]. The EB differentiates within the entry vacuole into metabolically active reticulate bodies (RB), which are non-infectious [2]. Infection is propagated further when the RBs differentiate back into EBs, which are released from the host cell by either exocytosis or lysis.

Inference by analogy with rodent models supports the existence of an effective immune response against *Chlamydia* bacteria [1]–[10]. These models of genital tract infections demonstrate that a large proportion of animals resolve primary infection and are temporarily resistant to reinfection [4]–[10]. When reinfection does occur, secondary inflammation and disease is significantly shorter, and bacterial load is greatly reduced.

One of the major immune mechanisms for controlling *Chlamydia* infection occurs through depletion of cellular tryptophan (TRP) by indoleamine-2,3-dioxygenase (IDO) – a T_H_1 process that is mediated by interferon gamma (IFN-*g*) [2], [11], [12]. A failed or weak T_H_1 response will allow *Chlamydia* RBs to respond to immune challenge by converting into a nonreplicating but revivable persistent state [12]. In this persistent state, chlamydia bacteria have been demonstrated to remain able to direct their own survival and still allow for antigen-presentation [11]. A direct consequence of this prolonged infection is antibody- or T_H_2-mediated hypersensitivity [12]. However, an over-stimulated T_H_1 response will lead to delayed-type hypersensitivity, and an increased risk of IFN-*g*-mediated tissue damage. Effective immune responses that successfully clear chlamydia infection will contain a proportional balance of cell-mediated and humoral immune responses [12].

With the exception of one early vaccine trial [13], equally-detailed insights into human immune responses have not extended beyond observational studies. However, elimination of infection is likely immune mediated [14]. Studies that have investigated the natural history of infections in humans have reported that resolution of chlamydia occurs within months, even years [15]–[17]. Previous estimates of the basic reproductive number (*R_0_*), in conjunction with epidemiological evidence from core groups, suggests that frequent exposure to *Ct* infections confers some degree of strain-specific protective immunity [18]. While these observed patterns are consistent with what might be produced by individual immune responses, it is interesting to note that they have not been directly measured [19]. As a result, this leaves some elementary questions unresolved. To our knowledge, little research has examined susceptibility to multiple chlamydial infections, explored the effect of exposure history on an individual's immune repertoire, or investigated any consequences for the spread of sexually transmitted *Ct* infections [4].

A person's history of exposure to STI-causing pathogens has been considered central to shaping their repertoire of effector B and T lymphocytes, as well as for driving STI persistence and evolution [19]–[20]. However, most, if not all, modeling related to bacterial STIs is concerned with the population dynamics of these infections. Although insightful, they often will disregard, or abridge any within-host dynamics. To better study the complexity of chlamydial infections within an infected individual, evaluate patient-centered interventions, and indentify future research priorities will require novel analytic tools [19], [21]–[22]. Our present objective, through numerical and analytic study of two within-host dynamic models, is to illuminate unique immunobiological characteristics that may result from differences in exposure history to chlamydia.

## Methods

**Figure 1:**
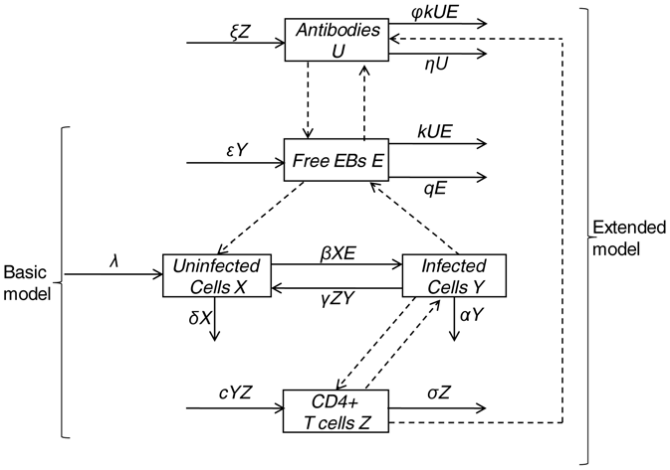
Stock and flow diagram illustrating the assumptions about chlamydial dynamics in the models. Displayed is a schematic representation of equations 1–5 in the main text. Uninfected ECs (*X*) are produced at a constant rate, *λ*, and die at a rate *δX*. Infected cells (*Y*) are produced at a rate *βXE* by contact with free EBs (*E*), and die at a rate *αY*. CD4+ T cells (*Z*) proliferate and differentiate at a rate *cY* and die at a rate *σZ*. Infected cells recover from infection at a rate *γZY*. Antibody (*U*) is produced at a rate *ξZ*. Antibodies decay at a rate *ηU*, neutralize EBs at a rate *kUE*, and are consumed in an EB-antibody complex at a rate 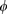 *kUE*. Basic and extended models are labeled by curved braces. Dashed arrows between state variables indicate interactions between them.

Our aim was to consider a highly parsimonious model and explore its dynamics in the context of repeated chlamydia infection. To do so, we constructed two simple mathematical models with explicit expressions for chlamydia bacteria, host target cells of the genital tract, and chlamydia-specific immune responses. The first, we termed a ‘basic’ model, and the second, an ‘extended’ model. These two structures represented a combination of dynamical hypotheses about chlamydia immunology. Throughout this study, they allowed us to iteratively investigate, and build confidence in, the role of both CD4+ T cell, and anti-chlamydia antibody responses under different exposure histories. Specific details of each model are discussed below, and schematically in Figure 1.

### A Basic Model of Within-Host Chlamydia Replication with CD4+ T cells

We elaborated a simple within-host model developed first by Nowak and Bangham [21]. Briefly, this model contains three variables that represent the population of uninfected cells, (*X*), infected cells, (*Y*), and cells of an intra-cellular pathogen (which, for the majority, have been viral particles), (*V*). Uninfected cells are produced at constant rate, *λ*, from a pool of precursor cells and die at a rate, *dX*. Infected cells are produced from uninfected cells and free virus at a rate *βXV* and die at a rate *aY*. Free virus is produced from infected cells at a rate *kY* and decline at a rate *uV*. Each of these quantities can denote the total abundance in a host, or the abundance in a given volume of blood or mass of tissue [23].

Because chlamydia bacteria are intracellular pathogens, the above model of viral dynamics was easily extended to model chlamydial dynamics. Our basic model included uninfected endothelial cells (ECs) of the genital tract (*X*),infected ECs (*Y*),free, infectious, and metabolically inactive EBs (*E*), T_H_1 CD4+ cells (*Z*). Uninfected ECs were produced at a constant rate, *λ*, and died at a rate *δX*. We used mass-action kinetics to model the interactions of uninfected cells with infectious EBs; infected cells were produced at a rate *βXE*, and died at a rate *αY*. We further assumed that activated chlamydia-specific CD4+ T cells proliferate and differentiate at a rate *cY*. In reality, the activation and proliferation of CD4+ T cells is induced by antigen-presenting cells; however here, we followed Nowak and May [23] in assuming that activation and proliferation is roughly proportional to the number of infected cells. Activated CD4+ cells died at a rate *σZ*.

The main effector mechanism of CD4+ T cells assumed in this model was IFN-*g*-mediated TRP starvation of chlamydial RBs. Here, the concentration of IFN-*g* and cytosolic TRP were modeled implicitly, and the concentration of each was assumed to be directly- and inversely proportional to the number of CD4+ cells, respectively. Because low levels of TRP have been demonstrated to have minimal effect on the viability of host cells [24], infected cells were therefore assumed to recover from infection at a rate *γZY*. These assumptions produced the set of ordinary differential equations:
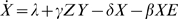
(1)

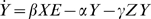
(2)

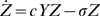
(3)

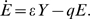
(4)


### An Extended Model of Within-Host Chlamydia Replication with CD4+ T cell and Antibody Responses

We extended the basic model to include chlamydia-specific antibody (*U*) that inhibit infection of genital tract ECs at a rate 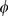 *kUE*. For this model, we ignored the effect of antibody-mediated cellular cytotoxicity because its significance during chlamydia infection has yet to be determined [10], [25]–[26]. We followed Yao et al [27] in assuming the production of antibody, *ξ*, is proportional to the number of CD4+ cells. Given that antigen-specific B and T cells are activated and proliferate in local lymphoid aggregate tissue in the genital tract by similar mechanisms [2], [27]–[28] our model assumed that they were roughly proportional to each other. For chlamydia, this appears to be a reasonable assumption for two reasons: the first because immune-mediated resolution requires a proportional balance of both T_H_1 and T_H_2 responses [11]–[12]; and the second because previous data suggest that the protective response of antibody is dependent upon CD4+ cells [10]. Previous studies that have explored this issue in sufficient detail for recurrent viral infections found negligible impacts on conclusions [27]. From Yao et al. [27], the natural decay rate of the antibody population was denoted by *η*, the efficacy of antibody-induced EB neutralization is denoted by *k*, and the number of antibody particles that are consumed in forming an EB-antibody complex is denoted by 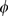.

Because our main interest is in the dynamics of the immune response under repeated infection, we included no explicit representation of the formation of memory T or B cells in either the basic or extended models. Instead, we model immune memory by letting our populations of immune cells (i.e., CD4+ T cells or antibody) die off slower than other cells following infection. There were two reasons for this: the first was because the results of the main study we were attempting to qualitatively reproduce did not explicitly measure populations of memory T cells [5]. Instead, T cell activity was reported an aggregated time series. This was later identified as CD4 T cell activity. As a direct result of this, we secondly thought it more appropriate to assume a heuristic approach over a mechanistic description of immune memory, since the end-result of immune memory (i.e., elevated levels of certain immune cells for some time after stimulation) is universal. These assumptions modified equation 4 of the basic model and produced an additional equation for antibody kinetics to form the extended model:
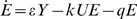
(4′)

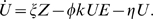
(5)Both models were solved numerically using the default Euler integration method in the modeling software Vensim DSS for Windows (version 5.5d).

Because these models are deterministic, CD4+ and antibody state variables cannot drive *Y*(*t*) or *E*(*t*) to zero. Therefore, following Wodarz et al. [29]–[30] we defined a threshold value below which chlamydia infection was considered extinct. Our extinction threshold was arbitrarily chosen to be marginally larger than the endemic equilibrium value of a model lacking a threshold, such that a single infection would eventually be cleared. We included an extinction threshold because we anticipated that this would better match empirical observations of individual infection and immune kinetics than a model without an extinction threshold. Initial conditions for the models were *X*(0) = 100, *Y*(0) = 0, *Z*(0) = 1, *E*(0) = 0.01, for both basic and extended models, and *U*(0) = 0.01, for the extended model.

Clearly, a complete mathematical description of the immune system is neither feasible nor analytically tractable, due to the vast complexity of the immune system. However, recent theoretical work has demonstrated the merit of simple mathematical models in reproduction and explanation of experimental results [29]–[33]. Our model structures were purposefully kept simple so to focus on broad immunobiological insights. The philosophy behind starting with a simple representation of a complex system designed to address certain well-defined questions is similar to that motivating the methods of experimental scientists [33]. Such simple models can often lead to important insights of a general nature into the factors or processes that shape epidemiological patterns [33].

### Parameterizing and Calibrating the Models to Experimental Data

Often, obtaining experimental estimates for many parameters in a model can be difficult [23], [27]. Where possible, however, the model has been parameterized to chlamydia-specific kinetics based on previous research [2]. Where no experimental or observational information could be readily obtained, the remaining parameters of the two within-host models were either taken from similar models [23], [27], or were calibrated to qualitatively approximate published experimental data from both mouse and guinea pig models [3]–[5], [7], [8], [10]. Specific parameter values are listed in Table 1. To ensure comparability between model and previous laboratory results (i.e., independence from particular units), all experimental and model results are presented non-dimensionally.

**Table 1:** Parameters for the basic and extended models.

Parameter	Description	Value (day^-1^)	References
*λ* ^§^	Rate uninfected cells restock.	1.54	25
*β* ^§^	Infection rate.	2.31	3-5,7-8,10
*δ*	Uninfected cell die-off.	0.01	3-5,7-8,10
*α*	Infected cell die-off.	0.01	3-5,7-8,10
*γ* ^§^	Rate infected cells are “cured” by CD4+ cells.	6.58	3-5,7-8,10
*c* ^§^	T cell responsiveness rate.	1.86	24
*σ*	T cell die-off.	0.02	3-5,7-8,10
*ε* ^§^	EB† production rate from infected cells.	2.02	2
*q*	Rate of EB decay.	2.00	3-5,7-8,10
ξ*^§^	Ab^‡^ production rate.	0.12	4,9-10
η*	Ab decay rate.	0.05	4,9-10
φ* ^§^	Number of Ab consumed per disabled EB.	5.41×10^-5^	4,9-10
k*	Rate EBs are disabled for each EB-Ab complex.	0.001	25

† EB  =  Elementary Body; ^‡^Ab  =  Antibody.

Parameters included in the extended model are indicated by (*). Parameters with (§) have been scaled to be dimensionless with respect to state variables. Scaling was based on an arbitrarily select reference category. State variable reference categories (*y_ref_*, *e_ref_*, *z_ref_*, and *u_ref_*) were the peak values (or lowest, for *x_ref_*) at initial infection.

### Re-exposure Scenarios

Because exposure history to, and subsequent reinfection with, chlamydia is of current epidemiological concern, we examined the long-term within-host dynamics under four different re-exposure scenarios. The first scenario was intentionally chosen because it mimicked protocol from previous experimental studies using guinea pig models. Here, a single re-exposure occurred approximately between 70 and 80 days after the resolution of a primary infection. The second investigated single re-exposure at regular intervals at 100, 200, 300, or 600 days after primary infection. The third scenario studied multiple re-exposures, every 30 days within 300 days of primary infection. The fourth was a combination of scenarios one and three (i.e., frequent re-exposure, though not as frequent as scenario three, within 300 days followed by a single re-exposure at 900 days after initial infection). The second through fourth re-exposure scenarios were chosen to be arbitrarily to aid in our understanding of the systems of equations would behave over longer time horizons. Re-exposure was modeled by an instantaneous inflow of EBs at each of the above-described times using a multiple of the initial infectious dose (i.e., 10×*E*(0)).

## Results

### Both Models Reproduce Experimentally-observed Kinetics of Primary and Secondary Chlamydial Infections

**Figure 2:**
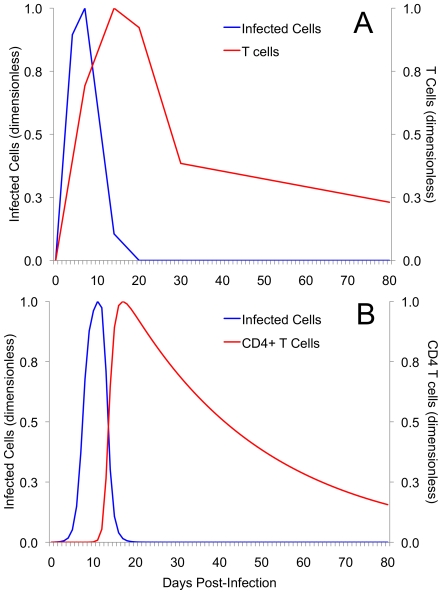
A comparison of experimental and simulated kinetics of primary chlamydia infection and CD4+ T cell responses. (A) Experimental data for infected cells and T cells at initial infection. Data are modified from Igietseme and Rank [5]. Female guinea pigs were infected with the same amount of Guinea Pig Inclusion Conjuntivitis (GPIC) chlamydia bacteria. (B) Simulated behavior of infected cells (*Y*) and CD4+ T cells (*Z*) for the basic model demonstrates similar qualitative behaviour when calibrated to approximate part (A). While both basic and extended models reproduced experimental behaviour, only the results of the basic model are displayed. For comparison of qualitative behaviour, both experimental and simulated data for infected cells and T cells have been scaled and are displayed non-dimensionally.

Figure 2 illustrates that previously observed kinetics of infected cells and CD4+ T cells (Figure 2A) can be approximated by the basic model (Figure 2B). Although it may be argued that our parameter values do not exactly reproduce experimental results, we do not feel that our results stray qualitatively from what would be expected in reality. The presence of qualitative, rather than quantitative, similarity does not diminish the value of the insights gained through careful analysis of mathematical models [30]. While not demonstrated here, a version of the extended model that assumed antibody as the sole immune response was unable to resolve primary infection. This further reaffirms that CD4+ T cells have a decisive role in resolving infection.

**Figure 3:**
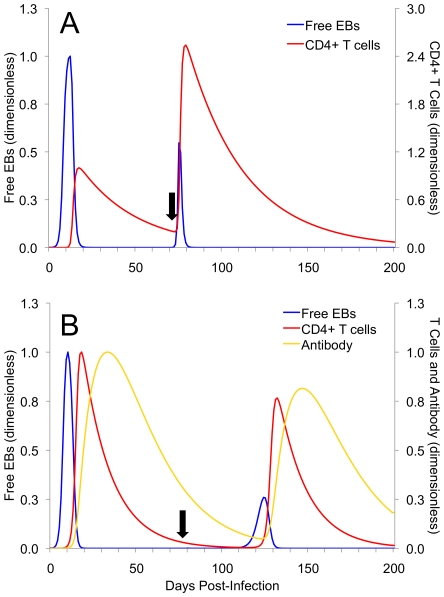
Secondary challenge experiments using the basic and extended models. Re-exposure was modeled by an instantaneous inflow of EBs at each of the above-described times using a multiple of the initial infectious dose, 10×*E*(0) for both the basic model (A), and the extended model (B). In both panels, second infection is decreased in severity because of some residual population of T cells (A) or both T cells and antibody (B). The magnitude and time evolution of the system of equations in both (A) and (B) have been rescaled (i.e., are presented non-dimensionally) so to allow for a visual comparison. Because of structural differences, the reference categories used for scaling were different between basic and extended models. Black arrow indicates time point of secondary exposure and CD4+ T cells and antibody are plotted on the right axis.

Figure 3 illustrates the behavior of the basic and extended models when exposure to a second chlamydia infection occurs. In both basic (Figure 3A) and extended (Figure 3B) models, a second infection is predicted to be of decreased magnitude. However, there are some interesting differences between the magnitudes of immune responses in each model: in Figure 3A, second infection at 75 days results in increased CD4+ cell activity compared to initial infection. These are common kinetics of free EBs and CD4+ T cell activity during a second infection in the absence of antibody [3]. In Figure 3B, reinfection at 75 days demonstrates a delay in positive growth of free EBs. While this is in contrast to the results of the basic model, it is this delay that supports the role of antibody in controlling reinfection [10].

### Increasing the Time between Initial and Second Infection increases Bacterial Load

**Figure 4:**
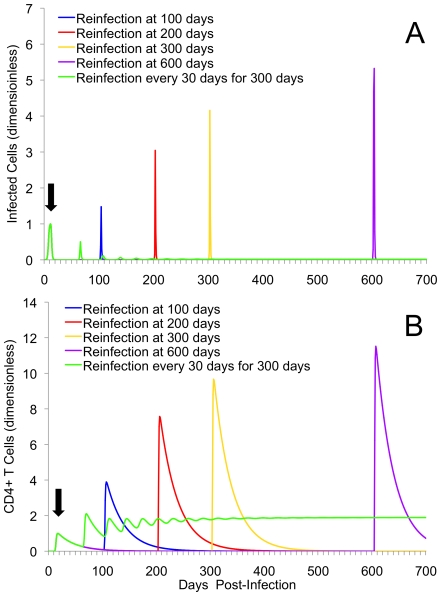
Multiple re-exposure experiments using the basic model. Displayed are the kinetics of infected cells (A), and CD4+ T cells (B) for single reinfection at either 100, 200, 300, or 600 days after initial infection in the basic model. Also included is the simulated kinetics of frequent re-exposure every 30 days over a span of 300 days after initial infection. As time of second infection occurs at increasingly long intervals from initial infection, the magnitude of infection and immune responses increases. For frequent re-exposure, it should be noted that oscillatory behavior continued after the removal of further infection. Single and multiple re-exposure scenarios are used to represent individuals that have low and high sexual exposure to chlamydia, respectively. Black arrow indicates point of initial infection (common to all scenarios). The magnitude and time evolution of the system of equations in both (A) and (B) are presented non-dimensionally.

**Figure 5:**
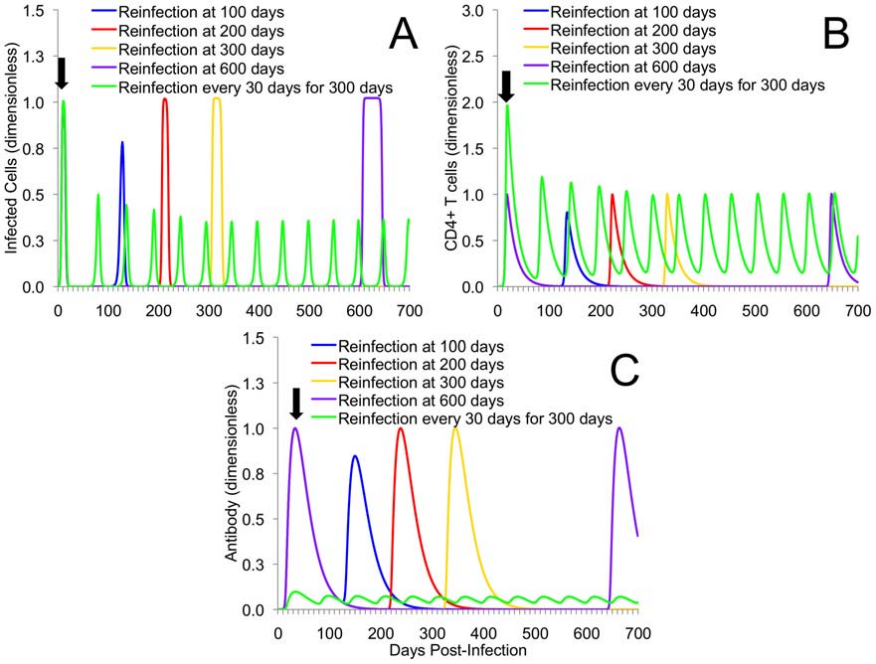
Multiple re-exposure experiments using the extended model. Displayed are the kinetics of infected cells (A), CD4+ T cells (B), and chlamydia-specific antibody (C) for single reinfection at either 100, 200, 300, or 600 days after initial infection in the extended model. Also included is the simulated kinetics of frequent re-exposure every 30 days over 300 days after initial infection under antibody deficiency. As time of second infection occurs at increasingly long intervals from initial infection, the magnitude of infection and immune responses increases. It should be noted that, for frequent re-exposure scenarios oscillatory behavior occurred once a deficiency in antibody is created, and they continue once infection pressure is removed. This demonstrates the dominance of a critical negative feedback antibody levels have on controlling reinfection. Black arrow indicates point of initial infection (common to all scenarios). For parts (B) and (C), initial immune cell levels are higher (part B) and lower (part C) at initial infection because of imposed antibody deficiency. The magnitude and time evolution of the system of equations in both (A), (B), and (C) are presented non-dimensionally.

In Figures 4 and 5 we examined a single re-exposure at increasingly long intervals from initial infection (re-exposure scenario 2). In the case of both models, similar dynamical behavior is observed: as the time between initial and secondary infection increases, the severity of that second infection also appears to increase. In the basic model this is associated with an increased magnitude of infected cells (Figure 4A). In the extended model, this was illustrated by an increased duration of elevated infected cells (Figures 5A).

Immune responses were also affected. For the basic model, reinfection at later times from initial infection appears to produce higher levels of CD4+ cells (Figure 4B). In the extended model, however, negative feedbacks associated with both antibody and CD4+ T cells keep immune levels within a range similar to that seen for initial infection (Figure 5B and 5C).

### Repeated Exposure Reinforces the Importance of Antibodies and Suggests a Role in Preventing Persistent Infections and Immunopathology

**Figure 6:**
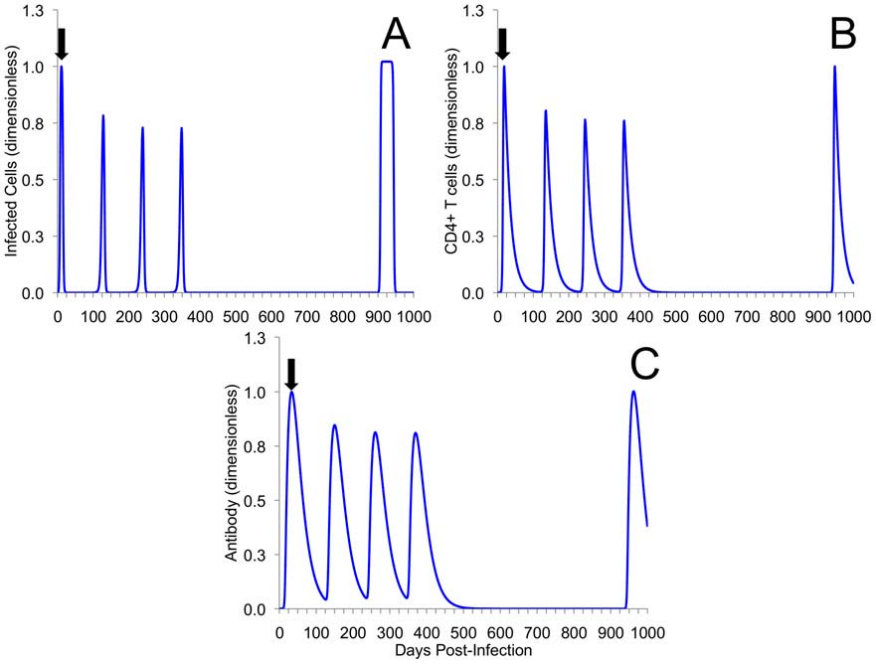
Higher initial re-exposure followed by a long period of no exposure. Displayed is the kinetics of infected cells (A), CD4+ T cells (B), and chlamydia-specific antibody (C) for the extended model with re-exposure every 100 days over a span of 300 days followed by single re-exposure 900 days after initial infection. With some level of pre-existing ‘immunity’ reinfection is demonstrated to be less severe. However, once infection pressure is removed, and immunity is allowed to wane, reinfection closely resembles initial infection. These scenarios were used to represent higher initial rates of exposure, between 100 and 300 days, followed by low exposure between 300 and 900 days. Black arrow indicates point of initial infection. The magnitude and time evolution of the system of equations in both (A), (B), and (C) are presented non-dimensionally.

In addition to repeated single re-exposure, Figures 4 and 5 also display frequent re-exposure, as might occur amongst commercial sex workers or core members of a sexual network (re-exposure scenario 3). Figure 6 contains the simulated outcomes for someone who is experiencing higher initial frequency of exposure, though not as high as scenario 3, followed by a long period of no exposure (re-exposure scenario 4). For EBs and infected cells in the basic model (Figure 4), frequent re-exposure every 30 days produced damped oscillations that approach a low endemic equilibrium that persists in the absence of further exposure. Given that CD4+ T cell responses are positively correlated with production of IFN-*g*, the basic model demonstrates that frequent re-exposure is likely to keep CD4+ cell population elevated, and the production of IFN-*g* continual.

For baseline values of the extended model, where an individual's antibody response is proportional to CD4+ cells, this behavior was not demonstrated (not shown). However, by lowering their antibody response, frequent re-exposure produces similar elevated predator-prey like immune responses and persistent infection that continued in the absence of further exposure (Figure 5). Repeated exposure, regardless of how frequently it occurs does not affect the behaviour of the models. In fact, the model's trajectories to both the endemic equilibrium (basic model) and the limit cycle (extended model) are conserved regardless of how frequently re-exposure occurs. An analytic study of re-exposure corroborates the simulated results for both basic and extended models (Supporting Information S1; Figure S1). Taken together, this suggests that a proportional antibody response may have an important role in preventing the formation of persistent infection. Figure 6 demonstrates that removing short-term frequent exposure to chlamydia allows partial immunity (i.e., immunity that does not prevent infection, but will reduce the duration and severity of second infection) to wane and return to near baseline levels.

## Discussion

Human immunobiology and reinfection rates have been the focus of recent debate in STI epidemiological literature [34]–[36]. From this debate, it is evident that inferences about longer-term immunobiological kinetics that are derived from short-term experimental research should be tentative. Our primary concern in this paper was to illuminate unique immunobiological characteristics that may result from differences in an individual's exposure history. Using two simple within-host models has allowed us to qualitatively explore some elementary questions about anti-chlamydial immunity under repeated exposure.

Overall, our results suggest several generalised interpretations that agree well with previously observed experimental studies: one, CD4+ T cell responses impart a marked level of immunity to primary and secondary infections; two, proportional antibody and CD4+ cell responses contribute in an important way to resolution of primary infection as well as an individual's immunity against reinfection; and three, when reinfection does occur after being re-exposed in relatively rapid succession (relative to the immunological decay time) the resulting infection is less severe and produces a decreased bacterial load. This latter result has provided significant insight into the timing of reinfection and the severity and duration of disease: as long as some level of pre-existing immunity remains, repeat infections are likely to be of decreased severity and duration when compared to initial infection. However, this does not appear to apply to repeat infections that occur at time points after immunity has waned further.

As mentioned previously, the major immune mechanisms for controlling *Ct* infection occurs through depletion of cellular TRP by IFN-*g*-inducible IDO [2], [11]–[12]. An effective immune response will contain a balance of cell-mediated and humoral immune responses [12]. A failed or weak T_H_1 response will allow for the development of antibody- or T_H_2-mediated hypersensitivity, whereas an over-stimulated T_H_1 response will lead to an increased risk of IFN-*g*-mediated tissue damage [12].

Our experiments of repeated exposure have highlighted two novel immunobiological outcomes that appear to connect the formation of antibody to the outcome infection and long-term protective immunity. Firstly, the in-host models used here suggest that for frequent exposure to chlamydia, the formation of a proportional antibody response (i.e., T_H_1 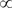 T_H_2) is not only central for preventing reinfection, but for regulating an individual's CD4+ T cell populations as well. Specifically, antibody will reduce the population of free EBs, which reduces the levels of infected cells; a lower magnitude of infected cells will regulate the activation and proliferation of CD4+ T cells, the production of IFN-*g*, and potentially lessen the risk of inflammatory damage. However, a lack of a proportional antibody response during frequent repeated infection allows for more infected cells to be produced, thus requiring higher levels of CD4+ T cells to eliminate infection.

Although T_H_1 CD4+ responses are positively correlated with the production of IFN-*g*, and an increased risk of inflammatory damage [14], our results indicate that the likelihood of persistent infection also increases. Because fluctuations between acute replication, IFN-*g*-mediated immune responses, and persistent infection are believed to the norm with chlamydia infections [12], our results outline a potentially previously unidentified mechanism of chronic infection that is driven by continual disproportionately high T_H_1 responses. To our knowledge, research into the link between persistent infection and immune responses has not assigned an important role to the prolonged activity to IFN-*g*-mediated mechanisms.

The second outcome demonstrates that the formation of chronic infection, in the presence of disproportionately high levels of T_H_1 CD4+ responses, also paradoxically appears to be associated with the development of complete immunity. Previous observational research using highly sexually active human populations (i.e., commercial sex workers) has reported that despite high prevalence rates of STIs [37], [38], some degree of protective long-term immunity may exist [18]–[19]. At a general level, the results from both our simple and extended models appear to support these previous observations: frequent re-exposure can yield stable and persistent elevated immune memory (Figures 4 and 5). It also appears that individuals experiencing brief periods of more-frequent rates of exposure (Figure 6) experience immunity that does not prevent infection, but will likely reduce the duration and severity of secondary infection and disease.

Although the accumulation of immunity through repeated infection has been assumed elsewhere [20], our results demonstrate that for those who would not have initially-frequent rates of exposure, removal of repeated exposure allows levels of immune cells to return to near baseline levels (Figure 6). Taken together, our results suggest that the natural development of complete, long-lived immunity to chlamydia may be the exception rather than the rule. This leads us to conclude that – for all but the most heavily exposed – both partial and complete immunity, in the long-term, may simply be an artifact of continued exposure coupled with disproportionate immune responses. At the very least, it appears that the formation of immune memory to chlamydia infection, among those frequently exposed, may not fit the standard precepts of infection and acquired immunity. This appears to challenge current evidence of chlamydial immunity and suggests the need for novel empirical data.

Because the models here are stylized descriptions of chlamydia infection and host immune responses, we have erred on the side of starting simple to help gain some initial insights into model dynamics. In particular, we chose to develop these within host models under simple assumptions about the spread of infection in the genital tract and the development of immune memory. For the spread of infection, we chose to develop a within-host model that describes populations of cells within the genital tract that interact in a mass-action like (or random mixing) process. While this is often assumed for most mathematical models of infectious disease spread, it could be argued that this is not a realistic assumption for the genital tract (i.e., it is not a homogenously-mixed system), and that our model does not capture events that occur within the genital tract. There are indeed better representations of an infection in the genital tract than those of mass-action. For example, we might have considered density-dependent, saturation-based, or spatial components as a model for infection spread. For the development of immune memory, we chose a heuristic approach over a mechanistic description. Because memory cells are activated at a faster rate than naive cells, and because they may proliferate before they acquire effector functions, the inclusion of state variable for immune memory would have altered the time course of our re-challenge experiments changed the dynamics of the results we have presented. As a result, the repeat exposures may have to be separated by a longer period of time to see the effects that were observed here.

Models are only as useful as the data available and parameter values estimated to inform them. It is important to recognize that, while our models were able to adequately reproduce the qualitative behaviour from laboratory results, they were calibrated without incorporating activation rates and proliferation rates of immune cells that have been published in previous experimental and mathematical studies [e.g., Refs 39]–[42]. While we have no doubt that these previously published papers are a valuable source of information, many focused their attention on primate models, or have been calibrated to very specific laboratory data that may not directly translate to the models we have used here. We are currently evaluating how the results of these papers may be applied to better inform our models. What is important to note is whether these models are able to shed light on specific questions in a way that alternative models, including human intuition, does not.

Despite the limitations discussed, with some modification these within-host models might be able to lend valuable insight towards two prominent, and less well-understood, characteristics of chlamydia infections [2]. The first is distinguishing immunobiological characteristics of symptomatic infection and asymptomatic infection; the second is estimating the likelihood of immunopathogenesis given either a persistent infection, or a number of previous chlamydial exposures. Collaborative efforts between ongoing modelling work and the collection of quantitative immunological information will be of great help determining important immunobiological thresholds for very specific clinical phenomena.

In general, control of STIs has been a remarkable yet incomplete public health achievement [19]. While a large amount of heterogeneity in the transmission of chlamydia has been attributed to variability human behaviour, immunobiological heterogeneity is also thought to influence its transmission [19]. A direct assessment of how immunobiology influences the transmission of chlamydia will require the use of theoretical tools that unify the features of immunology and sociology into an integrated ecological template [43]–[44]. Ideally, this will highlight any interdependence of chlamydia control programs and within-host immunobiology have on current epidemiological trends [45].

## Supporting Information

Supporting Information S1 This is the most recent version of the supporting information.
(0.05 MB DOC)

Figure S1 State space trajectory diagrams of CD4+ T cells versus Free Elementary Bodies for the basic and extended models during frequent re-exposure. For the basic model (A), re-exposure results in damped oscillations to an endemic equilibrium and high CD4+ T cell concentrations. However, in the extended model (B) re-exposure will produce a trajectory that approaches a stable limit cycle.
(0.50 MB TIFF)
