# Supplementary material for: Immunobiological Outcomes of Repeated Chlamydial Infection from Two Models of Within-Host Population Dynamics
Source: PLoS One. 2009 Sep 3;4(9):e6886. doi: 10.1371/journal.pone.0006886 (PMC2731222; doi:10.1371/journal.pone.0006886)
Supplement: Supporting Information S1 — This is the most recent version of the supporting information. (0.05 MB DOC) [file pone.0006886.s001.doc]

**Immunobiological Outcomes of Repeated Chlamydial Infection from Two Models of Within-host Population Dynamics – Supporting Information**

David M. Vickers, Qian Zhang, and Nathaniel D. Osgood

Existence and Stability of Fixed Points

This analytic study was performed to support the results of the simulation analyses. For initial infection (i.e., no re-exposure), the basic model has three equilibrium states. These include unstable disease- and ‘defense-free’ equilibria (, and , , , and $\hat{Z}=0$, respectively), and a locally stable endemic equilibrium: , $\hat{Y}=\frac{s}{c}$, , and . In the extended model, however, there exist five different fixed points. However, because two of them are non-physical (i.e., producing negative equilibrium values for state variables), and unreachable from initial conditions with non-negative state variables, we will only outline three of them. These included, as demonstrated in the basic model, disease- and ‘defense-free’ equilibria, as well as an endemic equilibrium. However in contrast to the basic model, none of these equilibria are stable and trajectories approach a limit cycle rather than a static equilibrium (see Figure S1).

Since the parameter values in each model were calibrated to produce chlamydia-specific results, they were held constant throughout the numerical simulations. This included the dose of chlamydial EBs at re-exposure. However, further investigation of our results suggests that a range of doses at re-exposure do not affect the stability of the fixed points in the basic model. More specifically, based on Routh-Hurwitz criteria we found that any non-negative perturbation to free EBs, *E* in the basic model will not change the stability of the endemic equilibrium [46]. A similar analysis in the extended model revealed that the endemic equilibrium will likely remain unstable for shorter-term re-exposure doses similar to that of initial infection. However, an interesting finding was that very large (compared to initial infection), prolonged exposure can render the endemic equilibrium stable.
