# Supplementary figures and images for: Immunobiological Outcomes of Repeated Chlamydial Infection from Two Models of Within-Host Population Dynamics
Source: PLoS One. 2009 Sep 3;4(9):e6886. doi: 10.1371/journal.pone.0006886 (PMC2731222; doi:10.1371/journal.pone.0006886)

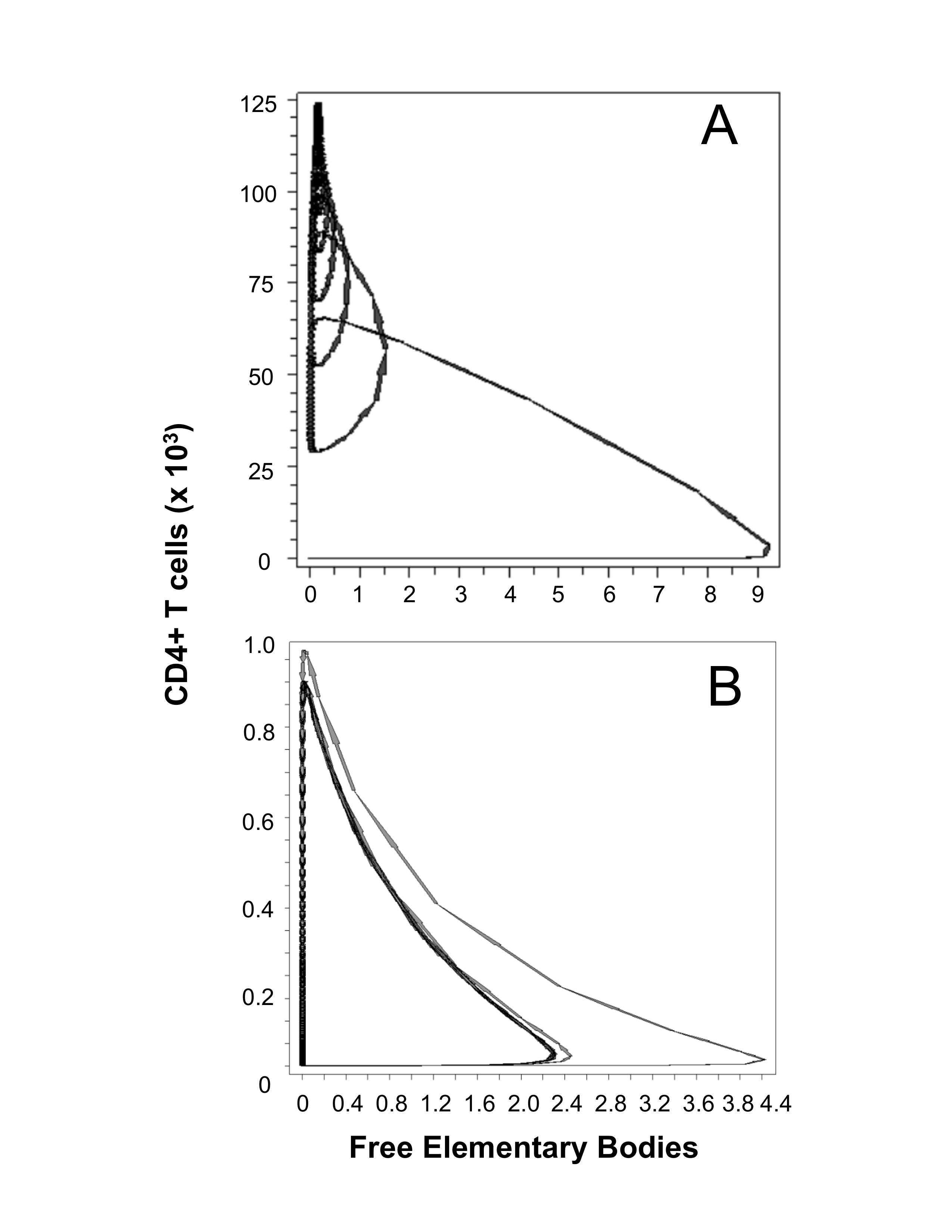

Supplement: Figure S1 — State space trajectory diagrams of CD4+ T cells versus Free Elementary Bodies for the basic and extended models during frequent re-exposure. For the basic model (A), re-exposure results in damped oscillations to an endemic equilibrium and high CD4+ T cell concentrations. However, in the extended model (B) re-exposure will produce a trajectory that approaches a stable limit cycle. (0.50 MB TIFF) [file pone.0006886.s002.tif]
